# Rauwolfia polysaccharide can inhibit the progress of ulcerative colitis through NOS2-mediated JAK2/STAT3 pathway

**DOI:** 10.1371/journal.pone.0301660

**Published:** 2024-04-16

**Authors:** Haidong Wu, Fan Jiang, Wei Yuan, Ye Zhao, Ning Liu, Xinpu Miao

**Affiliations:** 1 Department of Gastroenterology, Hainan General Hospital, Hainan Affiliated Hospital of Hainan Medical University, Haikou, Hainan, China; 2 Medical Centre for Digestive Diseases, The Second Affiliated Hospital of Nanjing Medical University, Nanjing, Jiangsu, China; 3 Department of the Center of Gerontology, Hainan General Hospital, Hainan Affiliated Hospital of Hainan Medical University, Haikou, Hainan, China; 4 Department of Emergency Surgery, Hainan General Hospital, Hainan Affiliated Hospital of Hainan Medical University, Haikou, Hainan, China; 5 Department of Gastroenterology, The First Affiliated Hospital of Zhengzhou University, Zhengzhou, Henan, China; 6 Department of Gastrointestinal Surgery, Hainan General Hospital, Hainan Affiliated Hospital of Hainan Medical University, Haikou, Hainan, China; University of Kansas Medical Center, UNITED STATES

## Abstract

**Background:**

Ulcerative colitis (UC) is an inflammatory disease of the digestive tract. Rauwolfia polysaccharide (Rau) has therapeutic effects on colitis in mice, but its mechanism of action needs to be further clarified. In the study, we explored the effect of Rau on the UC cell model induced by Lipopolysaccharide (LPS).

**Methods:**

We constructed a UC cell model by stimulating HT-29 cells with LPS. Dextran sodium sulfate (DSS) was used to induce mice to construct an animal model of UC. Subsequently, we performed Rau administration on the UC cell model. Then, the therapeutic effect of Rau on UC cell model and was validated through methods such as Cell Counting Kit-8 (CCK8), Muse, Quantitative real‑time polymerase chain reaction (RT-qPCR), Western blotting, and Enzyme-linked immunosorbent assay (ELISA).

**Results:**

The results showed that Rau can promote the proliferation and inhibit the apoptosis of the HT-29 cells-induced by LPS. Moreover, we observed that Rau can inhibit the expression of NOS2/JAK2/STAT3 in LPS-induced HT-29 cells. To further explore the role of NOS2 in UC progression, we used siRNA technology to knock down NOS2 and search for its mechanism in UC. The results illustrated that NOS2 knockdown can promote proliferation and inhibit the apoptosis of LPS-induced HT-29 cells by JAK2/STAT3 pathway. In addition, in vitro and in vivo experiments, we observed that the activation of the JAK2/STAT3 pathway can inhibit the effect of Rau on DSS-induced UC model.

**Conclusion:**

In short, Rauwolfia polysaccharide can inhibit the progress of ulcerative colitis through NOS2-mediated JAK2/STAT3 pathway. This study provides a theoretical clue for the treatment of UC by Rau.

## 1. Introduction

Ulcerative colitis (UC) is a form of inflammatory bowel disease (IBD) that begins in the rectum and extends in a continuous fashion, eventually involving part or the entire colon [1]. The lesions of the disease mainly invade the rectal mucosa and submucosa, forming erosions and ulcers. UC has two peak incidence patterns. The first peak occurs between the ages of 20 and 30, and the second peak occurs between the ages of 50 and 70 [2, 3]. In addition, the incidence of UC is reported to be higher in developed countries than in developing countries. In North America and Northern Europe, the incidence rate and prevalence rate of UC are the highest, with an incidence of 9–20 cases per 100,000 person-years and a prevalence of 156–291 cases per 100,000 people [4, 5]. The incidence of UC in Asian countries, including China, is also increasing year by year. In Hong Kong, the incidence of IBD tripled from 1.0 to 3.1 per 100,000 people [6]. Among them, Crohn’s disease, another inflammatory form of IBD, is more common than UC. In recent years, in the research progress of UC therapeutic drugs, traditional drugs such as 5-aminosalicylic acid, glucocorticoids and immunosuppressants have formed standardized treatment in the treatment plan [7–9]. Although there is a certain effect, the treatment effect is not ideal from the perspective of the tolerance of UC patients to the drug and the physiological health status after surgery [10].

Traditional Chinese medicine (TCM) is a conservative treatment for UC and is effective in relieving the symptoms of UC [11], primarily by regulating inflammatory cytokines and the immune system to protect the intestinal mucosa. Hainan Rauwolfia is a unique traditional Chinese medicine in Hainan and has a history of about 2000 years and is used to treat various human diseases [12]. It has the functions of clearing heat and detoxifying, anti-inflammatory and analgesic [13]. Moreover, Rauwolfia extract has been found to have an apparent inhibitory effect on prostate cancer [14], pancreatic cancer [15], ovarian cancer [16] and so on. The extract of Rauwolfia also has a therapeutic effect on colitis mice induced by DSS [17–19], but the mechanism needs to be further clarified.

In this study, we constructed a UC cell model by stimulating HT-29 cells with LPS and an animal model induced by DSS, and treated the model with Rau to explore the molecular mechanism of Rau in treating the UC model. This study provides a theoretical clue for the treatment of UC by Rau.

## 2. Methods

### 2.1. Chemicals and reagents

Rau (Customization, 5g) was obtained from Nanjing Daosifu Biotechnology Co., Ltd. LPS (L9143, 10mg) and DSS (60316ES25, 25g) was purchased from Sigma-Aldrich and Yeasen, respectively. Antibodies used in this study were as follows: iNOS (proteintech, 22226-1-AP), JAK2 (Affinity, AF6022), p-JAK2 (Abcam, ab195055), STAT3 (Zen-Bio, R22785), p-STAT3 (Cell signaling, 9145S), GAPDH (proteintech, 60004-1-Ig) and IgG (Zen-Bio, 511103). The IgG used for HE staining was also purchased from Zen-Bio (511103).

### 2.2 Cell culture

Human Colon Cancer Cell line HT-29 was obtained from iCell Bioscience Inc, Shanghai (iCell-h078). The cell line was cultured in McCoy’s 5A medium mixed with 10% fetal bovine serum and 1% penicillin-streptomycin. The cells were cultured in an incubator at 37°C, 5% CO_2_ and 95% air.

### 2.3 Establishment of rat model of ulcerative colitis

We purchased BALB/C mice from the First Affiliated College of Xi’an Jiaotong University. The mice were adaptively reared at 22–24°C and 20% humidity for one week and randomly divided into 4 groups (control group, DSS group, DSS+Rau group, DSS+Rau+colivelin group) with 9 mice in each group. The UC group mice were given 3% (w/v) DSS solution for free consumption for seven days, while the control group mice were fed with purified water for seven days. Rau(200mg/kg, 500μL) by gavage and colivelin(1mg/kg, 100μL) by intraperitoneal injection were administered daily for 7 days from the 8th day of modeling. The control and UC groups were given the same amount of distilled water. At 0d, 4d, 8d, 12d and 15d, the hair color, stool characteristics, stool occult blood and body weight of mice were observed and recorded. At the end of administration, the colon of the mice was dissected immediately after death, and the length of the colon was measured after laying it flat.

### 2.4 Cell proliferation

We completed cell proliferation by Cell Counting Kit-8 (MCE, HY-K0301). Firstly, HT-29 cells were inoculated into 96-well plates at 2×10^3^ cells/well and dealt with LPS at different concentration gradients (0μg/mL, 40μg/mL, 60μg/mL, 100μg/mL, 200μg/mL) to construct UC cell model. Different concentration gradients of Rau (50μg/mL, 100μg/mL, 200μg/mL, 400μg/mL) were used to treat the UC cell model. At 450nm wavelength, we detected the absorbance value of the above groups.

### 2.5 Cell apoptosis

Cell apoptosis was performed by cell apoptosis kit (Muse, MCH100105) based on the flow cytometer instrument. After cells were collected, we added Muse Annexin V & Dead Cell to the above groups. Then it was incubated at room temperature and shielded from light for 20min and tested on the machine. Finally, the cell apoptosis of each group was determined by calculating the sum of early and late apoptosis in each group.

### 2.6 RT-qPCR

Total RNA was extracted with Trizol from the collected cells. Nanodrop 2000 was used to detect RNA concentration and purity. RNA integrity was detected by 0.8% denaturing gel electrophoresis. Then, reverse transcribe 500ng RNA into cDNA with a TAKARA reverse transcription kit. The primers for the amplification of NOS2/JAK2/STAT3 cDNA were listed in Table 1. The RT-qPCR system included the primers, cDNA and SYBR High-Sensitivy qPCR SuperMix. Based on the fluorescence quantitative instrument (ABI 7500), we completed the PCR process and the PCR conditions were as follows: 1)1min at 95°C; 2) 45 cycles of 20s at 95°C, 20s at 60°C and 30s at 72°C; 3)4°C ∞.

**Table 1:** Primer sequence of NOS2/JAK2/STAT3.

Name	Forward primer (5’-3’)	Reverse primer (5’-3’)
NOS2	GCTCTACACCTCCAATGTGACC	CTGCCGAGATTTGAGCCTCATG
JAK2	CCAGATGGAAACTGTTCGCTCAG	GAGGTTGGTACATCAGAAACACC
STAT3	CTTTGAGACCGAGGTGTATCACC	GGTCAGCATGTTGTACCACAGG
GAPDH	GGAGCGAGATCCCTCCAAAAT	GGCTGTTGTCATACTTCTCATGG

### 2.7 Western blotting assay

After cells were collected, we extracted total protein by cell lysate containing protease inhibitors. Soon afterwards, we transferred them to sodium dodecyl sulfate-polyacrylamide gel (SDS-PAGE) for separation and then transferred them to polyvinylidene fluoride (PVDF) membranes. PVDF membranes were blocked with the blocking solution containing 5% non-fat milk and were incubated with primary antibodies at 4°C overnight. After cleaning the PVDF membrane with TBST, the membrane was incubated with the secondary antibody for 1h at room temperature. Finally, electrochemiluminescence (ECL) solutions were prepared in a dark chamber to visualize the target protein bands. The exposure time was ascertained in light of the fluorescence intensity of the target protein and the light intensity of the target protein was then analyzed using ImageJ.

### 2.8 ELISA

After 24h of incubation, the expression of IL-6 (JL20268), IL-1β (JL18442) and TNF-α (JL10484) in serum of colitis mice was detected by the enzyme-linked immunosorbent assay (ELISA) kit provided by Jianglai biology. Continuously measure the optical density (OD) of each well at 450nm using a multifunctional microplate reader (BioTek, Elx-800).

### 2.9 Disease activity index (DAI)

At 0d, 4d, 8d, 12d and 15d, the hair color, stool characteristics, fecal occult blood and body weight of the mice were observed and recorded. The DAI score was calculated according to the described method [20] to evaluate the severity of the disease.

### 2.10 HE staining

After the mice were killed at the end of the experiment, the whole colon was separated and rinsed with cold PBS, and then a part of the colon was fixed with 10% formalin and embedded in paraffin to make tissue sections. Subsequently, tissue sections were stained with hematoxylin and eosin according to the standard of practice [21] to assess the severity of colitis.

### 2.11 Statistical analysis

Data was expressed as mean ± standard deviation (SD). SPSS software 20.0 and GraphPad Prism 8.3.1 software were used to complete data collation and analysis. One-way analysis of variance was used for multiple group comparisons. *p*<0.05 indicates a statistically significant difference.

## 3. Results

### 3.1 Ulcerative colitis cell model was constructed by LPS-induced HT-29 cells

**Fig 1:**
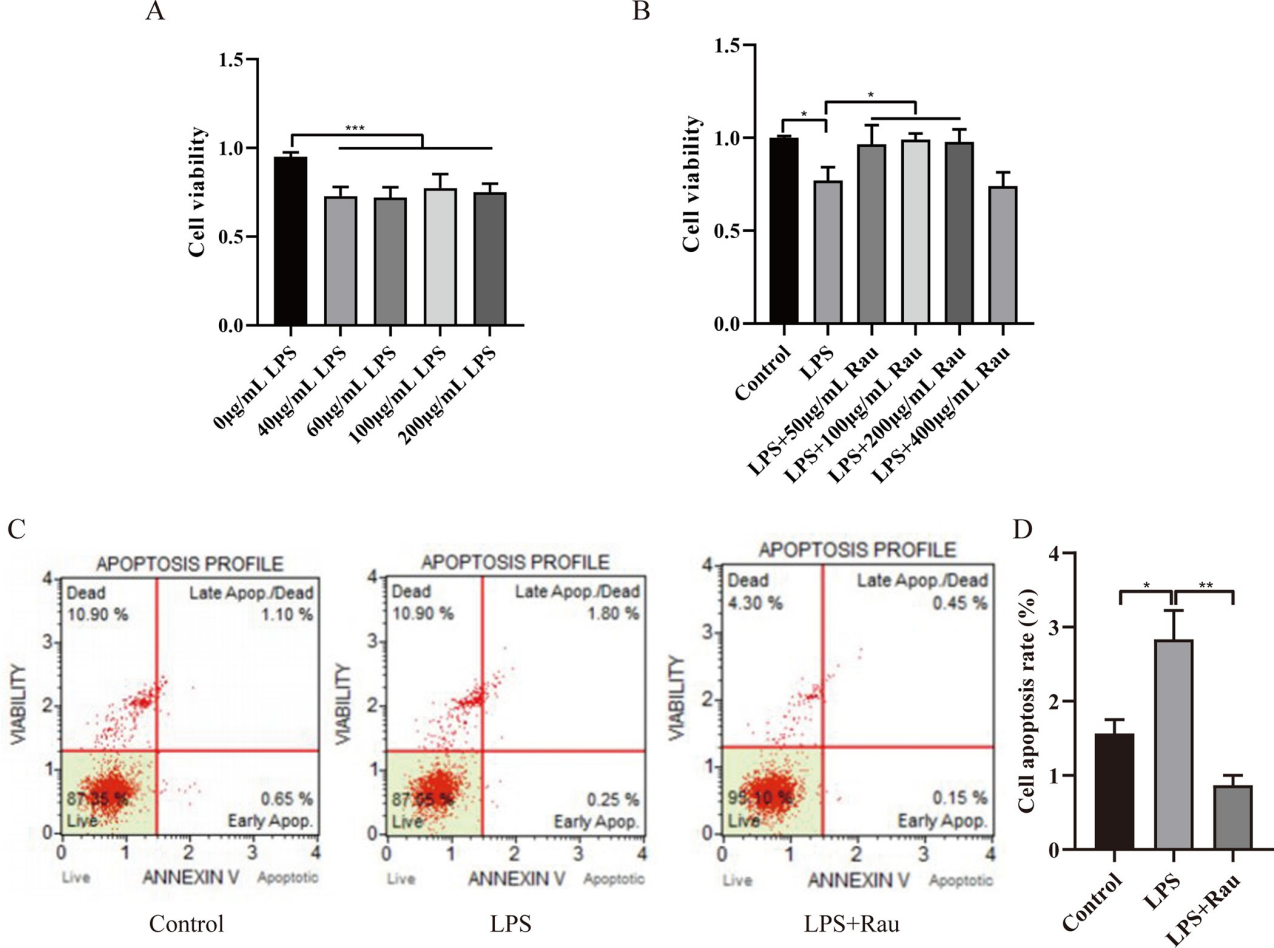
Rau can promote the proliferation and inhibit the apoptosis of the UC cell model induced by LPS. A: The cell viability of HT-29 cells treated with a series of concentrations of LPS (0μg/mL, 40μg/mL, 60μg/mL, 100μg/mL, 200μg/mL) was detected by CCK8. B: The cell viability of the UC cell model induced by LPS and treated with different concentrations of Rau (50μg/mL, 100μg/mL, 200μg/mL, 400μg/mL) was detected by CCK8. C: We observed cell apoptosis in the control group, the LPS group and the LPS+Rau group using Muse. D: The proportion of cell apoptosis in the control group, the LPS group and the LPS+Rau group was analyzed. The sum of the percentage of early apoptosis and the percentage of late apoptosis was the apoptosis ratio. *p < 0.05, **p < 0.01, ***p < 0.001.

We constructed a cell model of UC by stimulating HT-29 cells with a series of concentrations of LPS (0μg/mL, 40μg/mL, 60μg/mL, 100μg/mL, 200μg/mL). Firstly, the cell viability of each group was measured with the CCK8 kit. In **Fig 1A**, the cell viability of HT-29 cells decreased after treatment with various concentrations of LPS (all *p* < 0.001). LPS 60μg/mL was considered to be the optimal concentration, so this concentration was selected for the next experiment.

### 3.2 Rau can promote the proliferation and inhibit the apoptosis of LPS-induced HT-29 cells by NOS2/JAK2/STAT3 pathway

Studies have shown that the polysaccharides from Rau have anti-inflammatory effects, so we chose Rau to treat the UC cell model for molecular mechanism research. In **Fig 1B**, the toxicity of different concentrations of Rau (50μg/mL, 100μg/mL, 200μg/mL, 400μg/mL) on the model was detected by CCK8 kit. Compared with the group of LPS, low concentration of Rau (50μg/mL, 100μg/mL, 200μg/mL) can restore cell activity (all *p* < 0.05), while a high concentration of Rau (400μg/mL) can reduce cell viability. When the concentration of Rau was 100μg/mL, the cell viability of this group was comparable to that of the control group. Therefore, we chose Rau 100μg/mL to explore the following experiments.

In **Fig 1C**, we observed cell apoptosis of the control group, the LPS group and the LPS+Rau group, and the apoptosis ratio in each group was 1.75%, 2.05% and 0.60%, respectively. Soon afterwards, we conducted a statistical analysis of apoptosis in each group (**Fig 1D**). Compared with the control group, apoptosis was significantly increased in the LPS group (*p* = 0.033), and the addition of Rau can significantly reduce cell apoptosis (*p* = 0.004). The evidence indicated that Rau can promote the proliferation and inhibit the apoptosis of the UC cell model induced by LPS.

**Fig 2:**
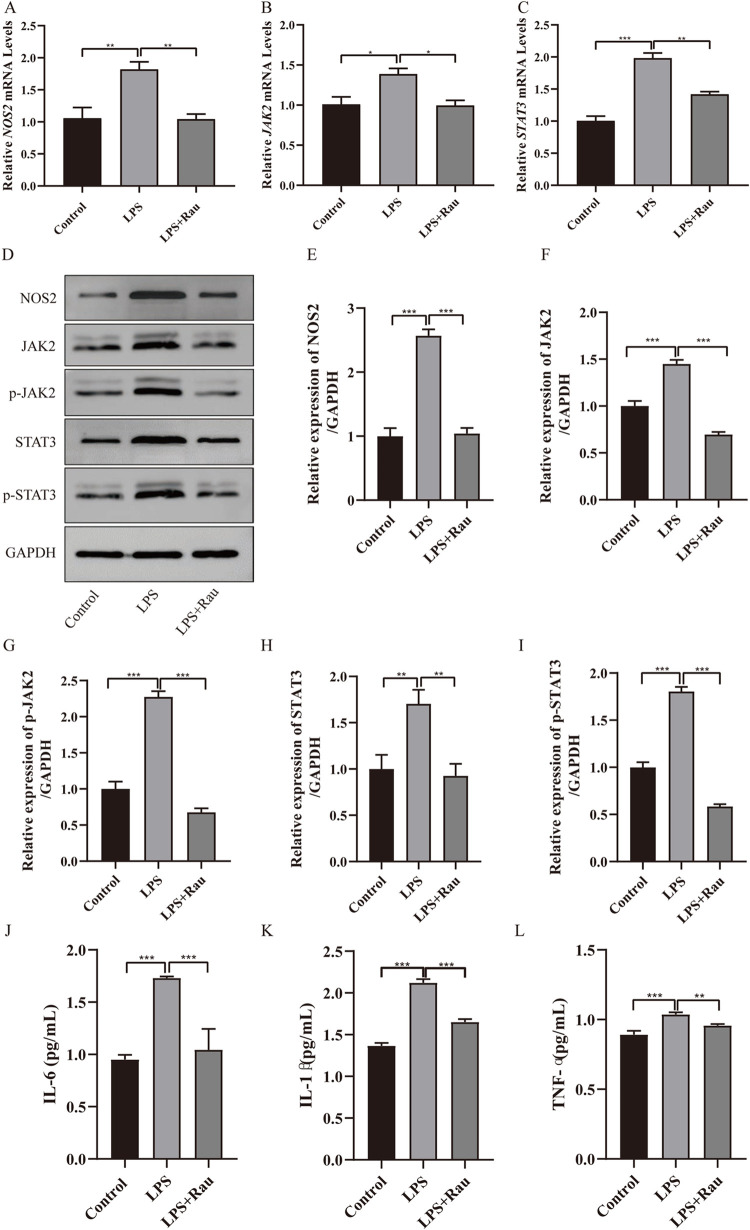
Rau can inhibit the expression of NOS2/JAK2/STAT3 and IL-6/IL-1β/TNF-α in UC cell model induced by LPS. A-C: The mRNA expression of *JAK2* and *STAT3* in the control group, the LPS group and the LPS+Rau group were detected by RT-qPCR. D: The protein expression of NOS2, JAK2, STAT3, p-JAK2 and p-STAT3 in the control group, the LPS group and the LPS+Rau group were detected by Western blotting assay. E-I: Perform grayscale analysis using Image J software. J-L: The protein expression of IL-6, IL-1β and TNF-αin the control group, the LPS group and the LPS+Rau group were detected by ELISA. Three independent samples were used for statistics. *p < 0.05, **p < 0.01, ***p < 0.001.

Moreover, we found that the mRNA expression of *NOS2*, *JAK2* and *STAT3* was notably increased by LPS stimulation (*p* = 0.003, *p* = 0.030, *p* < 0.001), while NOS2, JAK2 and STAT3 mRNA expression was significantly decreased after Rau was added (*p* = 0.003, *p* = 0.030, *p* = 0.003, **Fig 2A–2C)**. After LPS stimulation, the expression of NOS2, JAK2, p-JAK2, STAT3 and p-STAT3 was also significantly increased (*p* < 0.001, *p* = 0.001, *p* < 0.001, *p* = 0.003, *p* < 0.001). However, the protein expression of NOS2, JAK2, p-JAK2, STAT3 and p-STAT3 was reversed by the addition of Rau (*p* < 0.001, *p* < 0.001, *p* < 0.001, *p* = 0.002, *p* < 0.001), as shown in **Fig 2D–2I.** The results displayed that Rau can inhibit the expression of NOS2/JAK2/STAT3 in the UC cell model induced by LPS. What’s more, the expression of IL-6, IL-1β and TNF-α was also checked in the control group, the LPS group and the LPS+Rau group (**Fig 2J–2L**). The expression of IL-6, IL-1β and TNF-α in the LPS group was significantly higher than those in the control group (all *p* < 0.001). Nevertheless, the addition of Rau can inhibit the expression of IL-6, IL-1β and TNF-α induced by LPS (*p* < 0.001, *p* < 0.001, *p* = 0.005). The above results displayed that Rau can promote the proliferation and inhibit the apoptosis of LPS-induced HT-29 cells by NOS2/JAK2/STAT3 pathway.

### 3.3 NOS2 knockdown can promote proliferation and inhibit the apoptosis of LPS-induced HT-29 cells by JAK2/STAT3 pathway

**Fig 3:**
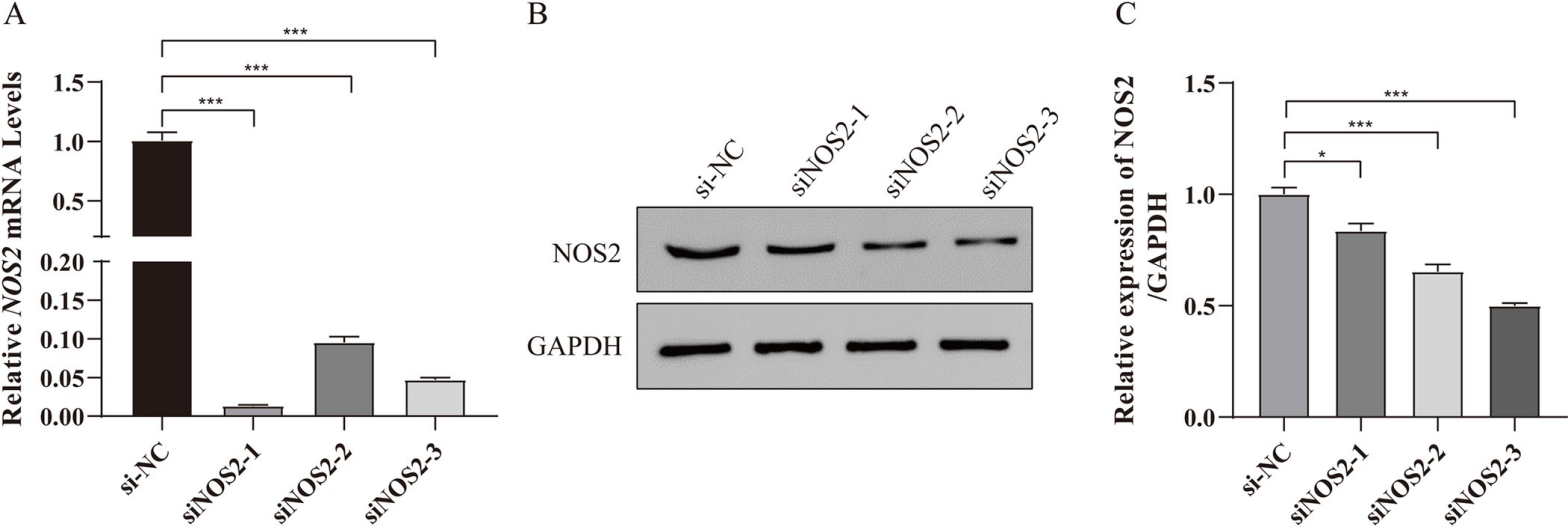
Screening NOS2 siRNA. A-B: After siNOS2-1, siNOS2-2 and siNOS2-3 transfected into the LPS-induced UC cell model, we tested the expression of NOS2 mRNA expression and NOS2 protein expression by RT-qPCR and Western blotting assay, respectively. C: Perform grayscale analysis using Image J software. Three independent samples were used for statistics. *p < 0.05, ***p < 0.001.

**Fig 4:**
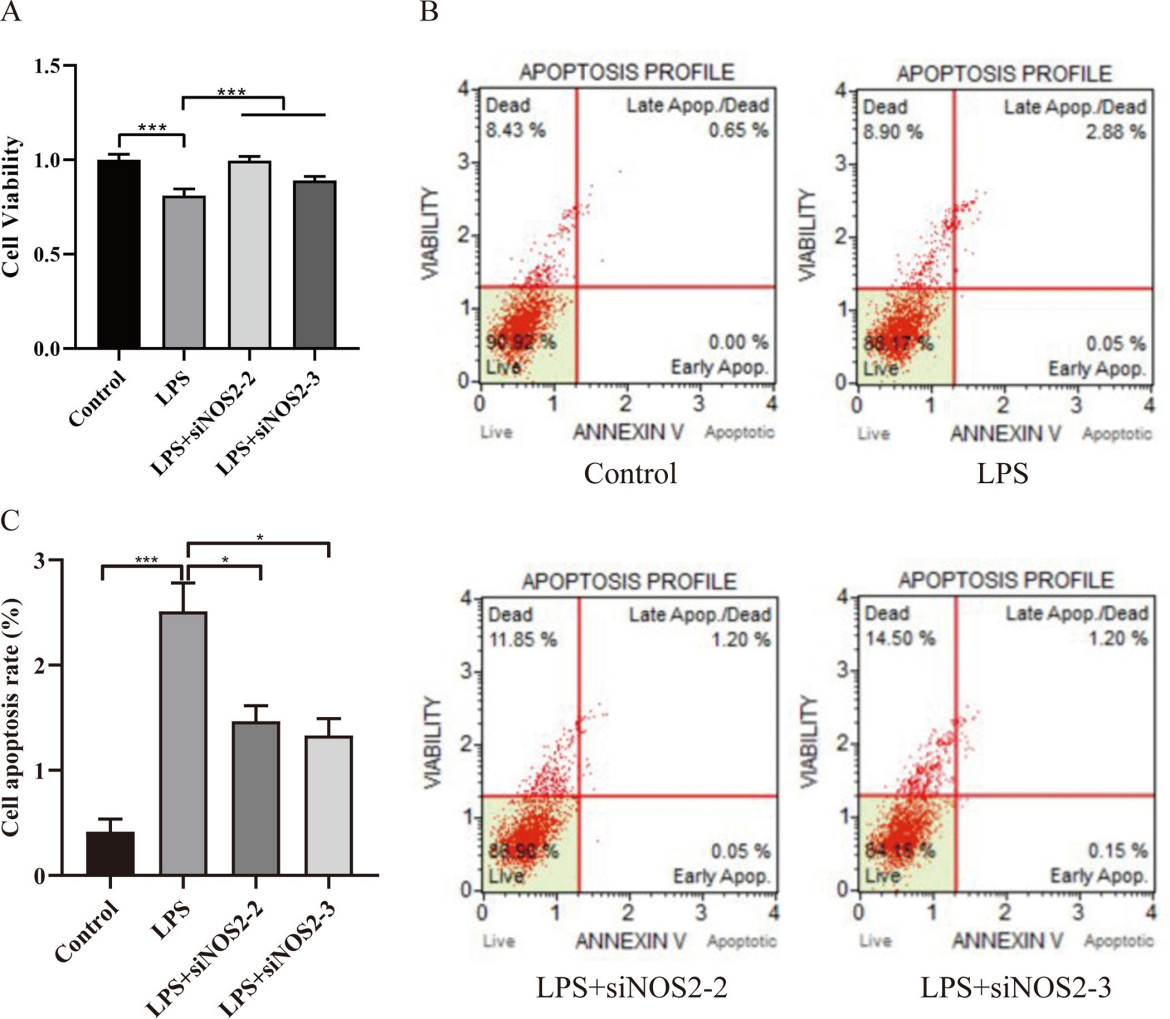
NOS2 knockdown can promote proliferation and inhibit the apoptosis of the UC cell model induced by LPS. A: We examined the effect of siNOS2-2 and siNOS2-3 on cell proliferation of LPS-induced UC cell model by CCK8. B: We examined the effect of siNOS2-2 and siNOS2-3 on cell apoptosis of LPS-induced UC cell model by Muse. C: The proportion of apoptosis in the control group, the LPS group, the siNOS2-2 group and the siNOS2-3 group was analyzed. The sum of the percentage of early apoptosis and the percentage of late apoptosis was the apoptosis ratio.

To further explore the role of NOS2 in LPS-induced ulcerative colitis, we designed and synthesized siNOS2-1, siNOS2-2 and siNOS2-3. In **Fig 3A–3C**, we tested the expression of NOS2 mRNA and NOS2 protein by RT-qPCR and western blot, respectively. The siRNA with the best knockdown effect was siNOS2-2 and siNOS2-3 (all *p* < 0.001). Next, we examined the effect of siNOS2-2 and siNOS2-3 on the proliferation and apoptosis of LPS-induced UC cell model (**Fig 4A–4C**). In **Fig 4A**, after the transfection of siNOS2, the proliferation ability of the LPS-induced UC cell model increased (all *p* < 0.001). Conversely, by contrast with the LPS group (apoptosis ratio: 2.93%), the apoptosis of cells transfected with siNOS2-2 and siNOS2-3 decreased (apoptosis ratio: 1.25% and 1.35%, respectively). The results showed that NOS2 knockdown can promote proliferation and inhibit the apoptosis of the UC cell model induced by LPS (**Fig 4B and 4C**).

**Fig 5:**
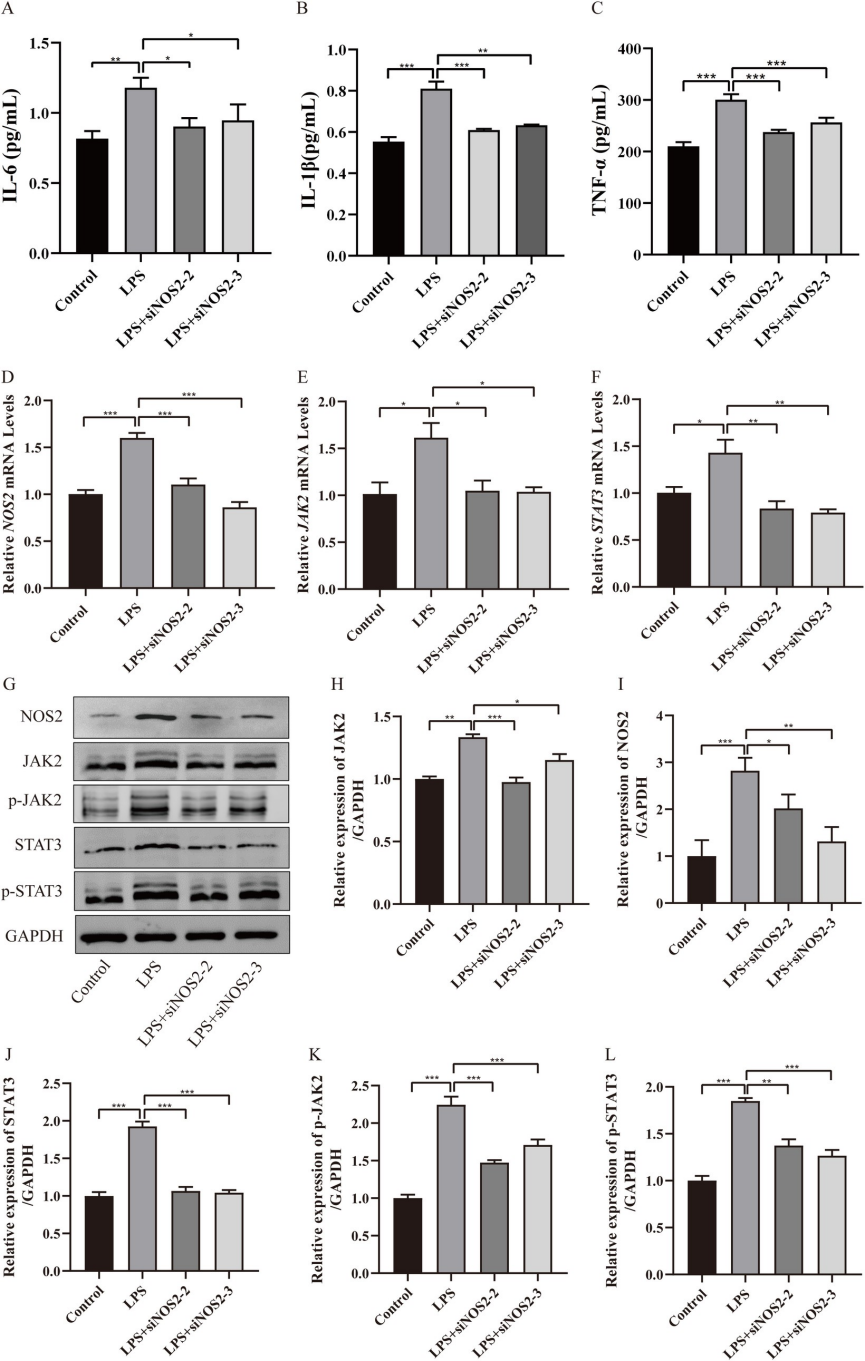
NOS2 knockdown can inhibit the expression of IL-6/IL-1β/TNF-α and JAK2/STAT3 in UC cell model induced by LPS. A-C: The protein expression of IL-6/IL-1β/TNF-α in the control group, the LPS group, the siNOS2-2 group and the siNOS2-3 group was detected by ELISA. D-F: The mRNA expression of *JAK2* and *STAT3* in the control group, the LPS group, the siNOS2-2 group and the siNOS2-3 group was detected by RT-qPCR. G: The protein expression of JAK2, STAT3, p-JAK2, p-STAT3 in the control group, the LPS group, the siNOS2-2 group and the siNOS2-3 group were detected by Western blotting assay. H-L: Perform grayscale analysis using Image J software. Three independent samples were used for statistics. *p < 0.05, **p < 0.01, ***p < 0.001.

Besides, we noticed that siNOS2-2 and siNOS2-3 reversed the expression of IL-6 (*p* = 0.011, *p* = 0.027), IL-1β (*p* < 0.001, *p* = 0.001) and TNF-α (all *p* < 0.001) in UC cell model induced by LPS (**Fig 5A–5C**). Meanwhile, we observed that siNOS2-2 and siNOS2-3 can inhibit the mRNA expression of *JAK2* (*p* = 0.036, *p* = 0.032) and *STAT3* (*p* = 0.006, *p* = 0.004) when compared with the LPS group (**Fig 5D–5F**). In comparison to the LPS group, siNOS2-2 and siNOS2-3 can significantly restrain the expression of JAK2 (*p* = 0.000, *p* = 0.022), STAT3 (*p* < 0.001, *p* < 0.001), p-JAK2 (*p* < 0.001, *p* < 0.001), p-STAT3 (*p* = 0.001, *p* < 0.001), as shown in **Fig 5G–5L**. From the above data, we speculated that Rau may promote proliferation and inhibit apoptosis of UC cell model through NOS2-mediated JAK2/STAT3 pathway.

### 3.4 Activation of JAK2/STAT3 pathway can inhibit the effect of Rau on LPS-induced HT-29 cells

**Fig 6:**
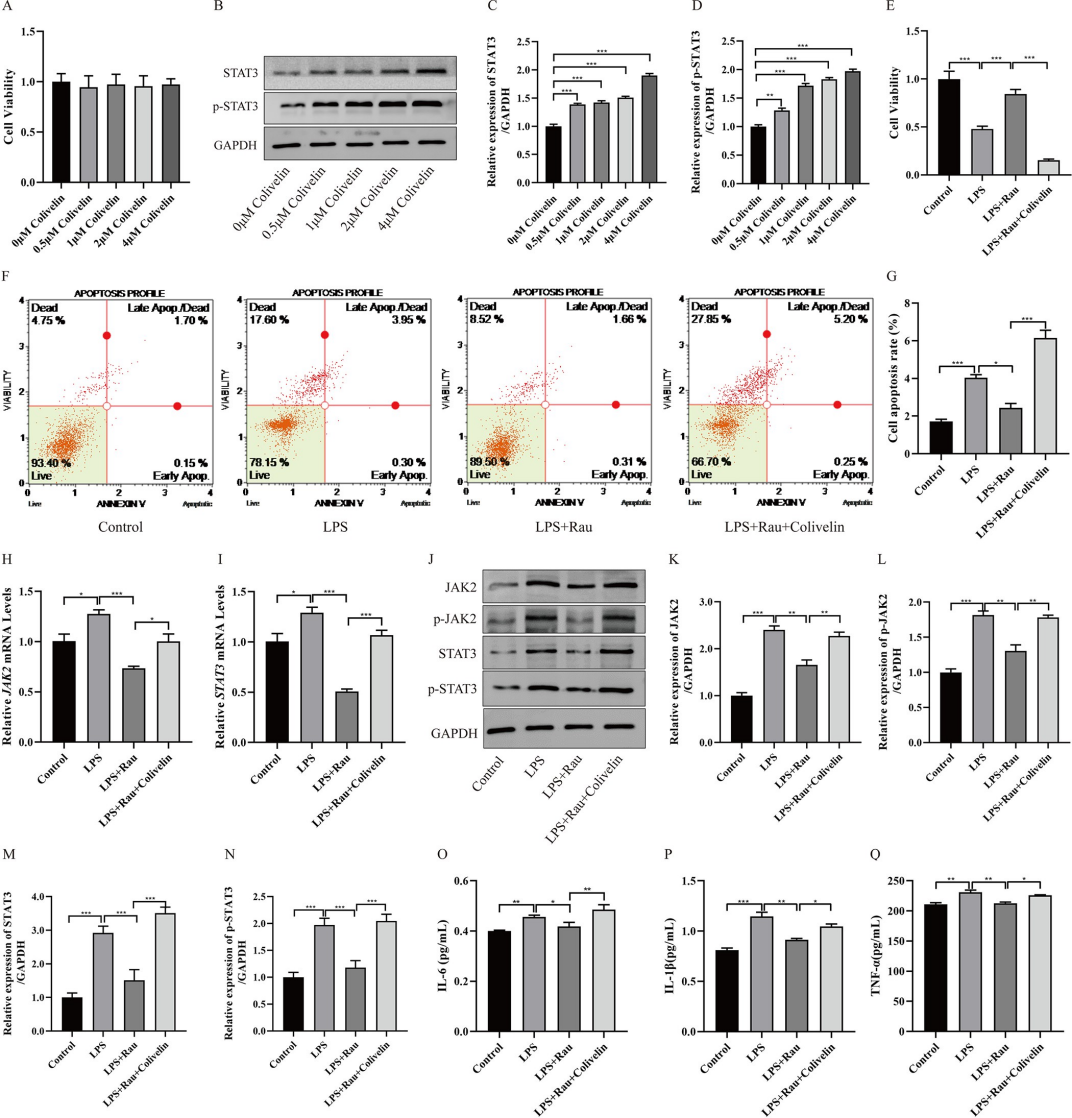
Activation of JAK2/STAT3 pathway can inhibit the effect of Rau on LPS-induced HT-29 cells. A: The UC cell model was treated with colvelin (0μM, 0.5μM, 1μM, 2μM, 4μM), the activator of JAK2/STAT3 pathway, and the cell activity was detected by CCK8. B: The UC cell model was treated with colvelin (0μM, 0.5μM, 1μM, 2μM, 4μM), and the protein expression level and phosphorylation level of STAT3 was detected by Western blotting assay. E: Cell proliferation in the control group, the LPS group, the LPS+Rau group and the LPS+Rau+colvelin group was detected by CCK8. F: Cell apoptosis in the control group, the LPS group, the LPS+Rau group and the LPS+Rau+colvelin group was detected by Muse. G-I: The mRNA expression of *JAK2* and *STAT3* in the control group, the LPS group, the LPS+Rau group and the LPS+Rau+colvelin group was detected by RT-qPCR. J: The protein expression of JAK2, STAT3, p-JAK2, p-STAT3 in the control group, the LPS group, the LPS+Rau group and the LPS+Rau+colvelin group was detected by Western blotting assay. C-D and K-N: Perform grayscale analysis using Image J software. O-Q: The protein expression of IL-6/IL-1β/TNF-α in the control group, the LPS group, the LPS+Rau group and the LPS+Rau+colvelin group was detected by ELISA. Three independent samples were used for statistics. *p < 0.05, **p < 0.01, ***p < 0.001.

To determine whether the effect of Rau on LPS-induced HT-29 cells was related to the JAK2/STAT3 pathway, we introduced the JAK2/STAT3 pathway activator-colivelin. First of all, we examined the effect of different concentrations (0μM, 0.5μM, 1μM, 2μM, 4μM) of colivelin on HT-29 cell viability, and the results showed that different concentrations of colivelin did not affect cell viability (**Fig 6A**). Also, as the concentration of colivelin increased, STAT3 and p-STAT3 expression increased significantly (**Fig 6B–6D**). According to the related articles [22, 23], we finally selected 1μM colivelin for follow-up experiments.

We tested the role of colivelin in LPS-induced HT-29 cells treated with Rau (**Fig 6E–6G**). In Fig 6E, the cell viability of the group of LPS+Rau+colivelin was significantly reduced in contrast to the group of LPS+Rau (*p* < 0.001). However, the apoptosis ratio in group of LPS+Rau and LPS+Rau+colivelin was 1.97% and 5.45%, respectively, illustrating that the cell apoptosis of LPS+Rau+colivelin group was significantly increased in contrast to LPS+Rau group (*p* < 0.001). These results suggested that colivelin can play a negative role in LPS-induced HT-29 cells treated with Rau.

Meanwhile, we estimated JAK2 and STAT3 expression level in the control, LPS, LPS+Rau and LPS+Rau+colivelin group (**Fig 6H–6N**). In **Fig 6H, 6I**, we observed that LPS+Rau stimulation can reduce the mRNA expression levels of JAK2 and STAT3, while the addition of colivelin can increase the levels (*p* = 0.038, *p* < 0.001). In **Fig 6J–6N**, in the LPS+Rau and LPS+Rau+colivelin group, we observed the same upward trend. Compared to the LPS+Rau group, JAK2 and STAT3 protein expression level were rising in the LPS+Rau+colivelin group (*p* = 0.036, *p* < 0.001). The phosphorylation level of JAK2 and STAT3 protein were also increased (*p* = 0.002, *p* < 0.001). These data confirm that JAK2/STAT3 pathway was involved in LPS-induced HT-29 cells treated with Rau. And activation of JAK2/STAT3 pathway can have a negative effect on LPS-induced HT-29 cells treated with Rau. In **Fig 6O–6Q**, the expression of IL-6, IL-1β and TNF-α was significantly increased (the LPS+Rau group vs. the LPS+Rau+colivelin group, *p* < 0.001, *p* = 0.033, *p* = 0.026). This result confirmed the negative effect of JAK2/STAT3 pathway activation in LPS-induced HT-29 cells treated with Rau.

### 3.5 Activation of the JAK2/STAT3 pathway can inhibit the effect of Rau on DSS-induced UC mice model

**Fig 7:**
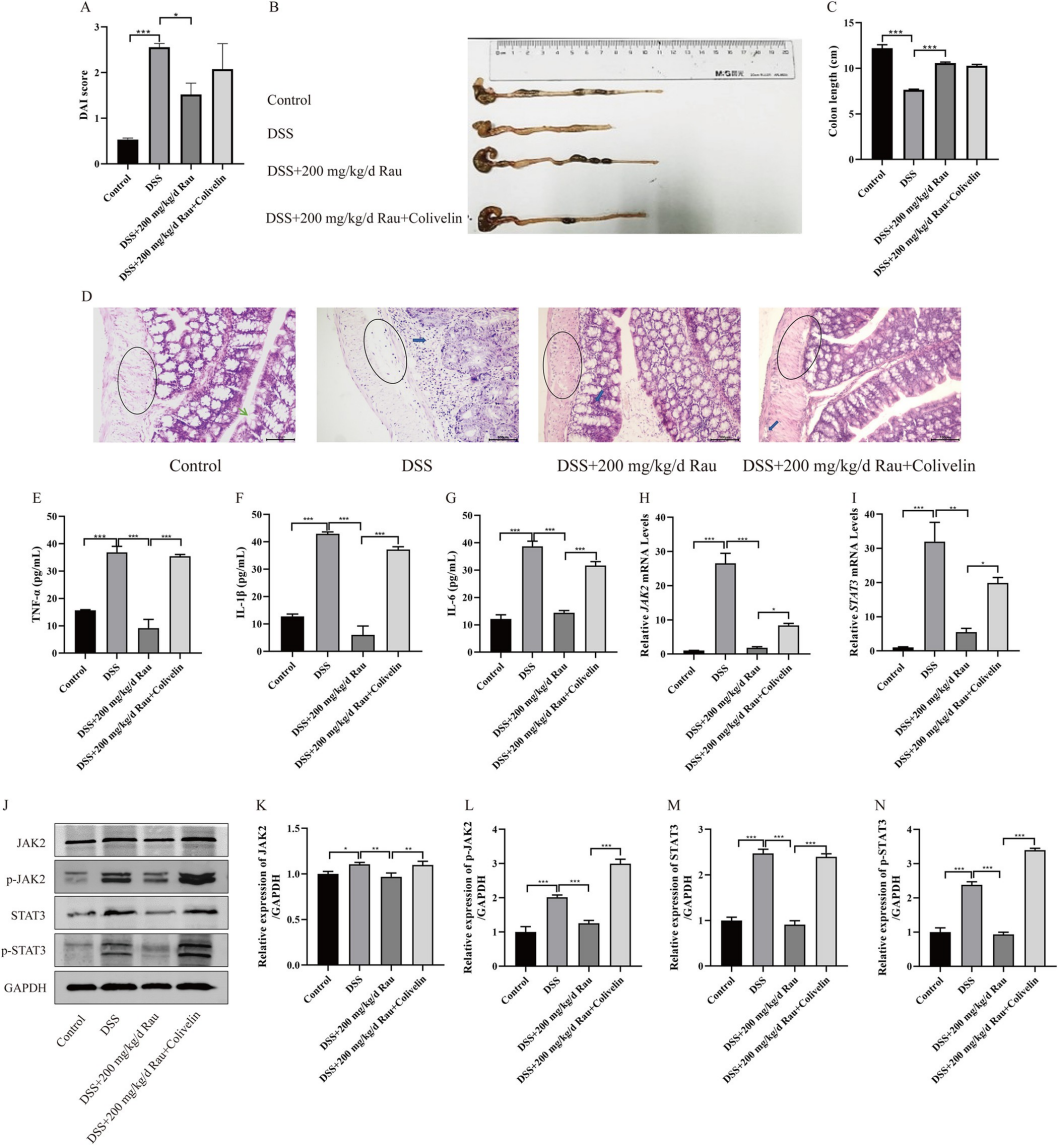
Activation of the JAK2/STAT3 pathway can inhibit the effect of Rau on DSS-induced UC mice model. A: DAI score of the control group, the DSS group, the DSS+200mg/kg/d Rau group and the DSS+200mg/kg/d Rau+colvelin group was calculated. B: Colon length of the control group, the DSS group, the DSS+200mg/kg/d Rau group and the DSS+200mg/kg/d Rau+colvelin group was calculated by ruler. C: The comparison results of colon length of the control group, the DSS group, the DSS+200mg/kg/d Rau group and the DSS+200mg/kg/d Rau+colvelin group were compared. D: Inflammatory reaction was detected in the control group, the DSS group, the DSS+200mg/kg/d Rau group and the DSS+200mg/kg/d Rau+colvelin group by HE. Blue circle: mucosal layer; Red circle: goblet cells; Green scissors: Inflammatory cells. E-G: The protein expression of IL-6/IL-1β/TNF-α in the control group, the DSS group, the DSS+200mg/kg/d Rau group and the DSS+200mg/kg/d Rau+colvelin group was detected by ELISA. H-I: The mRNA expression of *JAK2* and *STAT3* in the control group, the DSS group, the DSS+200mg/kg/d Rau group and the DSS+200mg/kg/d Rau+colvelin group was detected by RT-qPCR. J: The protein expression of JAK2, STAT3, p-JAK2, p-STAT3 in the control group, the DSS group, the DSS+200mg/kg/d Rau group and the DSS+200mg/kg/d Rau+colvelin group was detected by Western blotting assay. K-N: Perform grayscale analysis using Image J software. Three independent samples were used for statistics. *p < 0.05, **p < 0.01, ***p < 0.001.

In vivo, we used DSS to construct UC mice model to investigate the role of JAK2/STAT3 pathway activation in LPS-induced UC cell model treated with Rau. By observing the hair color, stool character, stool occult blood of DSS-induced UC mice model, and recording the change of body weight, the DAI score was calculated. In **Fig 7A**, DSS significantly increased the DAI score of mice (*p* < 0.001), while Rau inhibited DAI score of mice (*p* = 0.015). On the contrary, in **Fig 7B and 7C**, in the presence of 200 mg/kg/d Rau, the reduction in colon length induced by DSS in UC mice was greatly restored (*p* < 0.001). Unfortunately, the addition of colivelin did not significantly change the DAI score and colon length of the UC mice (*p* = 0.202, *p* = 0.744).

Besides, we observed that Rau can significantly reduce the degree of colon mucosal tissue separation, intestinal epithelial tissue injury and inflammatory cell infiltration in DSS-induced UC mice (**Fig 7D**). And in **Fig 7E–7G**, Rau inhibited the protein expression of TNF-α/IL-1β/IL-6 (*p* < 0.001, *p* < 0.001, *p* = 0.002) in DSS-induced UC mice. Similarly, Rau can inhibit the mRNA expression of JAK2/STAT3 (*p* = 0.002, *p* = 0.001) and the protein expression of JAK2, p-JAK2, STAT3, p-STAT3 (*p* = 0.005, *p* < 0.001, *p* < 0.001, *p* < 0.001) in DSS-induced UC mice (**Fig 7H–7N**). However, in the presence of colivelin, we observed that it can aggravate the degree of inflammatory damage-mild separation of colonic mucosa, injury of intestinal epithelium, infiltration of inflammatory cells in the mucosa (**Fig 7D**). It can also suppress the protein expression of TNF-α/IL-1β/IL-6 in serum of colitis mice (*p* < 0.001, *p* < 0.001, *p* = 0.003), the mRNA expression of JAK2/STAT3 (*p* = 0.028, *p* = 0.038), the protein expression of JAK2/STAT3 (*p* = 0.007, *p* < 0.001) and the phosphorylation level of JAK2/STAT3 (*p* < 0.001, *p* < 0.001) in colonic tissue of DSS-induced UC mice treated by Rau (**Fig 7H–7N**). Generally, Rau significantly alleviated the symptom of UC mice, but the activation of the JAK2/STAT3 pathway can inhibit the effect of Rau on DSS-induced UC mice.

## 4. Discussion

UC is an inflammatory disease of the digestive tract, mainly located in the colon and rectum, with a chronic, lifelong, and easily recurrent course. Due to the lack of specific measures to treat UC and its serious complications, the World Health Organization has designated it as a difficult disease to treat in modern times [24]. Although researchers have been studying for many years, they have not yet discovered a clear cause of UC. In the study, we observed that NOS2 knockdown can promote proliferation and inhibit the apoptosis of LPS-induced HT-29 cells by JAK2/STAT3 pathway. In addition, based on the LPS-induced UC cell model and DSS-induced UC mice model, the activation of the JAK2/STAT3 pathway was observed to inhibit the effect of Rau on UC model. Therefore, we speculate that Rau can inhibit the progress of ulcerative colitis through NOS2-mediated JAK2/STAT3 pathway.

NOS2 encodes inducible nitric oxide synthase, which can catalyze the production of nitric oxide, thus contributing to antibacterial and anti-pathogen activity [25]. Some researchers reveal that the mRNA expression and protein expression of NOS2 in UC samples are significantly up-regulated than that in control subjects [26, 27]. These data indicated that NOS2 may take part in the development of UC. And, selective NOS2 may have therapeutic promise in the treatment of UC [28]. Wei et al found that NOS2 gene is a key gene in the treatment of UC by Gegen Qinlian Decoction through network pharmacology and molecular docking technology [29]. In addition, in an article reported by Zhang et al [30], compared with the DSS-induced colitis mouse model, NOS2 expression in the colon tissue of mice was significantly decreased after administration of protoberberine rhizocorrhizine. The results showed that NOS2 may be a key target for UC development as well as for TCM treatment of UC. In our research, we found that Rau can inhibit the expression of NOS2 in the UC cell model induced by LPS, illustrating that NOS2 was the target for Rau treatment of UC. To further explore the role of NOS2 in UC progression, we used siRNA technology to knock down NOS2 and search for its mechanism in UC. In the present study, siNOS2 can inhibit the mRNA expression of *JAK2* and *STAT3* and significantly restrain the expression of JAK2, STAT3, p-JAK2, and p-STAT3. The results displayed that NOS2 knockdown can inhibit the expression of JAK2/STAT3 in the UC cell model induced by LPS. Therefore, we suppose that we speculated that Rau may promote proliferation and inhibit apoptosis of UC cell model through NOS2-mediated JAK2/STAT3 pathway, yet, further studies are warranted.

JAK2/STAT3 signaling pathway is one of the main signaling pathways of inflammation and is responsible for the production of cytokines, as well as the recruitment and activation of cytokines [31, 32]. Numerous studies have shown that JAK2/STAT3 pathway is an important pathway inflammation influence colitis. Recently, blocking the JAK2/STAT3 pathway can regulate immune responses and alleviate chronic inflammation of the intestine [33]. Wu et al adopt this approach to confirm that butyric acid enhances the therapeutic effect of EHLJ7 on ulcerative colitis by inhibiting the JAK2/STAT3 signaling pathway [34]. Furthermore, researchers have revealed that the anti-inflammatory mechanism of traditional Chinese medicine is related to the blocking of JAK2/STAT3 signaling pathway [35]. To further test this idea, we used JAK2/STAT3 activator to inversely verify that the failure of Rau’s anti-inflammatory mechanism in UC progress was associated with activation of the JAK2/STAT3 signaling pathway. We observed that the activation of the JAK2/STAT3 pathway can inhibit the effect of Rau on DSS-induced UC model.

In conclusion, the above data demonstrated that Rau can inhibit the progress of ulcerative colitis through NOS2-mediated JAK2/STAT3 pathway. In future, it is necessary for Rau to regulate UC models through NOS2/JAK2/STAT3 in further experiments.

## Supporting information

S1 Fig Flow diagram. (JPG)

S1 Raw image (ZIP)

## References

[1] Regueiro MD: Diagnosis and treatment of ulcerative proctitis. Journal of clinical gastroenterology 2004, 38(9):733–740. doi: 10.1097/01.mcg.0000139178.33502.a3 15365396

[2] Loftus EV Jr, Silverstein MD, Sandborn WJ, Tremaine WJ, Harmsen WS, Zinsmeister AR: Ulcerative colitis in Olmsted County, Minnesota, 1940–1993: incidence, prevalence, and survival. Gut 2000, 46(3):336–343. doi: 10.1136/gut.46.3.336 10673294 PMC1727835

[3] Mak WY, Zhao M, Ng SC, Burisch J: The epidemiology of inflammatory bowel disease: East meets west. Journal of gastroenterology and hepatology 2020, 35(3):380–389. doi: 10.1111/jgh.14872 31596960

[4] Talley NJ, Abreu MT, Achkar JP, Bernstein CN, Dubinsky MC, Hanauer SB et al: An evidence-based systematic review on medical therapies for inflammatory bowel disease. The American journal of gastroenterology 2011, 106 Suppl 1:S2–25; quiz S26. doi: 10.1038/ajg.2011.58 21472012

[5] Ordás I, Eckmann L, Talamini M, Baumgart DC, Sandborn WJ: Ulcerative colitis. Lancet (London, England) 2012, 380(9853):1606–1619. doi: 10.1016/S0140-6736(12)60150-0 22914296

[6] Ng SC, Tang W, Ching JY, Wong M, Chow CM, Hui AJ et al: Incidence and phenotype of inflammatory bowel disease based on results from the Asia-pacific Crohn’s and colitis epidemiology study. Gastroenterology 2013, 145(1):158–165.e152. doi: 10.1053/j.gastro.2013.04.007 23583432

[7] Ahmad A, Ansari MM, Mishra RK, Kumar A, Vyawahare A, Verma RK et al: Enteric-coated gelatin nanoparticles mediated oral delivery of 5-aminosalicylic acid alleviates severity of DSS-induced ulcerative colitis. Materials science & engineering C, Materials for biological applications 2021, 119:111582. doi: 10.1016/j.msec.2020.111582 33321628

[8] Xu X, Xu X, Ciren Y, Feng B, Tao C, Xia Y et al: Chemopreventive effects of 5-amino salicylic acids on inflammatory bowel disease-associated colonic cancer and colonic dysplasia: a meta-analysis. International journal of clinical and experimental medicine 2015, 8(2):2212–2218. 25932153 PMC4402800

[9] Bruscoli S, Febo M, Riccardi C, Migliorati G: Glucocorticoid Therapy in Inflammatory Bowel Disease: Mechanisms and Clinical Practice. Frontiers in immunology 2021, 12:691480. doi: 10.3389/fimmu.2021.691480 34149734 PMC8209469

[10] Hu Q, Tang XZ, Liu F, Liu DW, Cao B: Vedolizumab subcutaneous formulation maintenance therapy for patients with IBD: a systematic review and meta-analysis. Therapeutic advances in gastroenterology 2023, 16:17562848231166227. doi: 10.1177/17562848231166227 37124368 PMC10141260

[11] Liu Y, Li BG, Su YH, Zhao RX, Song P, Li H et al: Potential activity of Traditional Chinese Medicine against Ulcerative colitis: A review. Journal of ethnopharmacology 2022, 289:115084. doi: 10.1016/j.jep.2022.115084 35134488

[12] Lobay D: Rauwolfia in the Treatment of Hypertension. Integrative medicine (Encinitas, Calif) 2015, 14(3):40–46. 26770146 PMC4566472

[13] Liu PH, Liu YY, Chen D, Xu QQ, Wang CN: Study on anti-inflammatory effects of wild southern medicine Rauwolfia hainanensis, vol. 17; 2011.

[14] Bemis DL, Capodice JL, Gorroochurn P, Katz AE, Buttyan R: Anti-prostate cancer activity of a beta-carboline alkaloid enriched extract from Rauwolfia vomitoria. International journal of oncology 2006, 29(5):1065–1073. 17016636

[15] Yu J, Chen Q: Antitumor Activities of Rauwolfia vomitoria Extract and Potentiation of Gemcitabine Effects Against Pancreatic Cancer. Integrative cancer therapies 2014, 13(3):217–225. doi: 10.1177/1534735414532010 24764328

[16] Yu J, Ma Y, Drisko J, Chen Q: Antitumor Activities of Rauwolfia vomitoria Extract and Potentiation of Carboplatin Effects Against Ovarian Cancer. Current therapeutic research, clinical and experimental 2013, 75:8–14. doi: 10.1016/j.curtheres.2013.04.001 24465036 PMC3898180

[17] Yuan W, Tian Y, Lin C, Wang Y, Liu Z, Zhao Y et al: Pectic polysaccharides derived from Hainan Rauwolfia ameliorate NLR family pyrin domain-containing 3-mediated colonic epithelial cell pyroptosis in ulcerative colitis. Physiological genomics 2023, 55(1):27–40. doi: 10.1152/physiolgenomics.00081.2022 36440907

[18] Miao XP, Sun XN, Cui LJ, Cao QF, Zhuang GF, Deng TZ et al: Suppressive effect of pectic polysaccharides extracted from Rauwolfia verticillata (Lour.) Baill.var.hainanensis Tsiang on inflammation by regulation of NF- κ B pathway and interleukin-17 in mice with dextran sulphatesodium-induced ulcerative colitis. Asian Pacific journal of tropical medicine 2015, 8(2):147–152. 25902030 10.1016/S1995-7645(14)60306-0

[19] Cui LJ, Yuan W, Chen FY, Wang YX, Li QM, Lin C et al: Pectic polysaccharides ameliorate the pathology of ulcerative colitis in mice by reducing pyroptosis. Annals of translational medicine 2022, 10(6):347. doi: 10.21037/atm-22-877 35434032 PMC9011308

[20] Chaudhary G, Mahajan UB, Goyal SN, Ojha S, Patil CR, Subramanya SB: Protective effect of Lagerstroemia speciosa against dextran sulfate sodium induced ulcerative colitis in C57BL/6 mice. American journal of translational research 2017, 9(4):1792–1800. 28469784 PMC5411927

[21] Dai Y, Lu Q, Li P, Zhu J, Jiang J, Zhao T et al: Xianglian Pill attenuates ulcerative colitis through TLR4/MyD88/NF-κB signaling pathway. Journal of ethnopharmacology 2023, 300:115690. 36075274 10.1016/j.jep.2022.115690

[22] Xu T, Wu K, Shi J, Ji L, Song X, Tao G et al: LINC00858 promotes colon cancer progression through activation of STAT3/5 signaling by recruiting transcription factor RAD21 to upregulate PCNP. Cell death discovery 2022, 8(1):228. doi: 10.1038/s41420-022-00832-w 35468892 PMC9038718

[23] Chen Y, Hou L: HCRP-1 alleviates the malignant phenotype and angiogenesis of oral squamous cell carcinoma cells via the downregulation of the EGFR/STAT3 signaling pathway. Oncology letters 2022, 24(5):387. doi: 10.3892/ol.2022.13507 36276496 PMC9533360

[24] Feakins RM: Ulcerative colitis or Crohn’s disease? Pitfalls and problems. Histopathology 2014, 64(3):317–335. doi: 10.1111/his.12263 24266813

[25] Dhillon SS, Mastropaolo LA, Murchie R, Griffiths C, Thöni C, Elkadri A et al: Higher activity of the inducible nitric oxide synthase contributes to very early onset inflammatory bowel disease. Clinical and translational gastroenterology 2014, 5(1):e46. doi: 10.1038/ctg.2013.17 24430113 PMC3912315

[26] Wang H, Zhang R, Wen S, McCafferty DM, Beck PL, MacNaughton WK: Nitric oxide increases Wnt-induced secreted protein-1 (WISP-1/CCN4) expression and function in colitis. Journal of molecular medicine (Berlin, Germany) 2009, 87(4):435–445. doi: 10.1007/s00109-009-0445-4 19238344

[27] Rafa H, Benkhelifa S, AitYounes S, Saoula H, Belhadef S, Belkhelfa M et al: All-Trans Retinoic Acid Modulates TLR4/NF-κB Signaling Pathway Targeting TNF-α and Nitric Oxide Synthase 2 Expression in Colonic Mucosa during Ulcerative Colitis and Colitis Associated Cancer. Mediators of inflammation 2017, 2017:7353252. 10.1155/2017/7353252 PMC5376956 28408791

[28] Kankuri E, Hämäläinen M, Hukkanen M, Salmenperä P, Kivilaakso E, Vapaatalo H et al: Suppression of pro-inflammatory cytokine release by selective inhibition of inducible nitric oxide synthase in mucosal explants from patients with ulcerative colitis. Scandinavian journal of gastroenterology 2003, 38(2):186–192. doi: 10.1080/00365520310000681 12678336

[29] Wei M, Li H, Li Q, Qiao Y, Ma Q, Xie R et al: Based on Network Pharmacology to Explore the Molecular Targets and Mechanisms of Gegen Qinlian Decoction for the Treatment of Ulcerative Colitis. BioMed research international 2020, 2020:5217405. doi: 10.1155/2020/5217405 33299870 PMC7710413

[30] Zhang JL, Zhang MN, Wang HG, Yang XZ, Yu CG: Jatrorrhizine alleviates ulcerative colitis via regulating gut microbiota and NOS2 expression. Gut pathogens 2022, 14(1):41. doi: 10.1186/s13099-022-00514-z 36271438 PMC9587631

[31] Bousoik E, Montazeri Aliabadi H: "Do We Know Jack" About JAK? A Closer Look at JAK/STAT Signaling Pathway. Frontiers in oncology 2018, 8:287. doi: 10.3389/fonc.2018.00287 30109213 PMC6079274

[32] Roskoski R Jr.: Janus kinase (JAK) inhibitors in the treatment of inflammatory and neoplastic diseases. Pharmacological research 2016, 111:784–803. doi: 10.1016/j.phrs.2016.07.038 27473820

[33] Fernández-Clotet A, Castro-Poceiro J, Panés J: Tofacitinib for the treatment of ulcerative colitis. Expert review of clinical immunology 2018, 14(11):881–892. doi: 10.1080/1744666X.2018.1532291 30285500

[34] Tang X, Li X, Wang Y, Zhang Z, Deng A, Wang W et al: Butyric Acid Increases the Therapeutic Effect of EHLJ7 on Ulcerative Colitis by Inhibiting JAK2/STAT3/SOCS1 Signaling Pathway. Frontiers in pharmacology 2019, 10:1553. doi: 10.3389/fphar.2019.01553 32038241 PMC6987075

[35] Jiang M, Zhong G, Zhu Y, Wang L, He Y, Sun Q et al: Retardant effect of dihydroartemisinin on ulcerative colitis in a JAK2/STAT3-dependent manner. Acta biochimica et biophysica Sinica 2021, 53(9):1113–1123. doi: 10.1093/abbs/gmab097 34259316

