# Supplementary figures and images for: Rauwolfia polysaccharide can inhibit the progress of ulcerative colitis through NOS2-mediated JAK2/STAT3 pathway
Source: PLoS One. 2024 Apr 16;19(4):e0301660. doi: 10.1371/journal.pone.0301660 (PMC11020939; doi:10.1371/journal.pone.0301660)

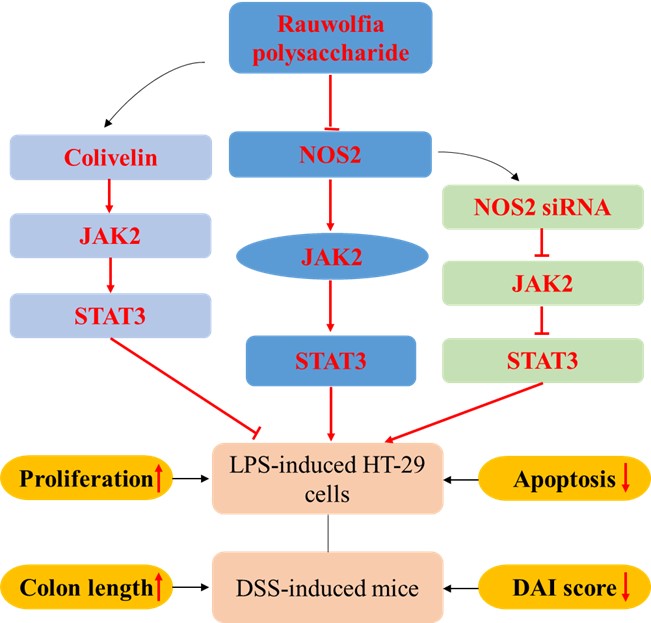

Supplement: S1 Fig — (JPG) [file pone.0301660.s001.jpg]
