# Supplementary material for: Rauwolfia polysaccharide can inhibit the progress of ulcerative colitis through NOS2-mediated JAK2/STAT3 pathway
Source: PLoS One. 2024 Apr 16;19(4):e0301660. doi: 10.1371/journal.pone.0301660 (PMC11020939; doi:10.1371/journal.pone.0301660)
Supplement: S1 Raw image — (ZIP) [file pone.0301660.s002.zip › S1_raw_images.pdf]

GAPDH 36 kDa

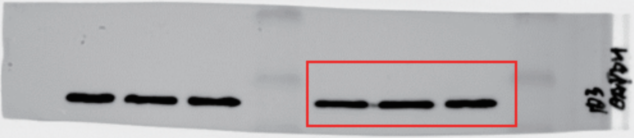

X X X Control LPS LPS+Rau

NOS2 131 kDa

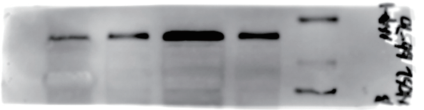

X Control LPS LPS+Rau

JAK2 131kD

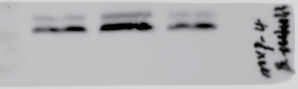

Control LPS LPS+Rau

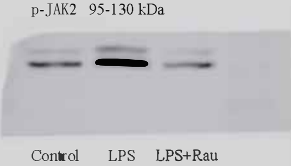
p-JAK2 95-130 kDa
Control
LPS
LPS+Rau

STAT3 88 kDa

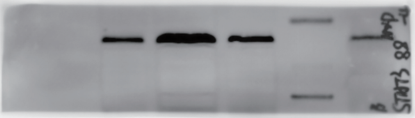

Control LPS LPS+Rau X

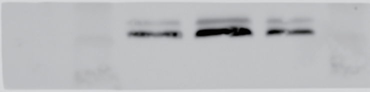
p-STAT3 80-100 kDa
Control
LPS
LPS+Rau

GAPDH 36 kDa

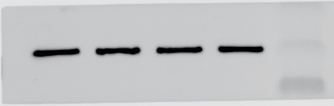

si-NC siNOS2-1 siNOS2-2 siNOS2-3

NOS2 131 kDa

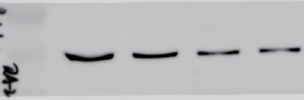

GAPDH 36 kDa

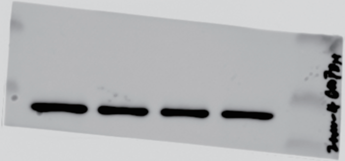

Control LPS LPS+siNOS2-2 LPS+siNOS2-3

NOS2 131kD

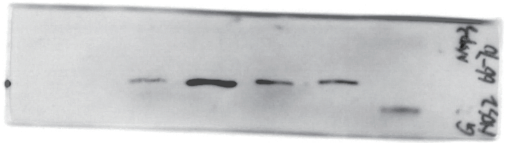

Control LPS LPS+siNOS2-2 LPS+siNOS2-3

JAK2 131kD

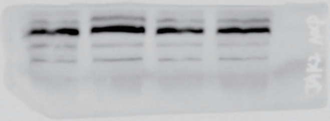

Control LPS LPS+siNOS2-2 LPS+siNOS2-3

p-JAK2 95-130 kD

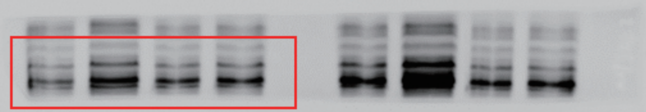

Control LPS LPS+siNOS2-2 LPS+siNOS2-3 X X X X

p-STAT3 80-100 kDa

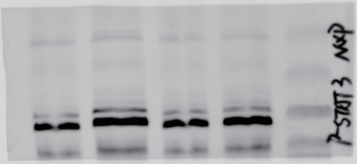

Control LPS LPS+siNOS2-2 LPS+siNOS2-3

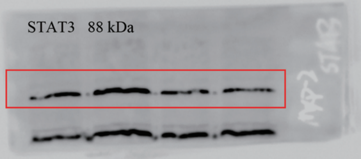

Control LPS LPS+siNOS2-2 LPS+siNOS2-3

GAPDH 36 kDa

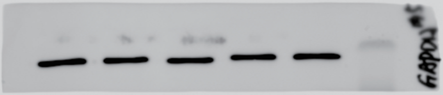

0uM Colivelin 0.5uM Colivelin 1uM Colivelin 2uM Colivelin 4uM Colivelin

p-STAT3 80-100 kDa

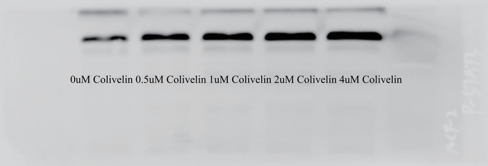

STAT3 88 kDa

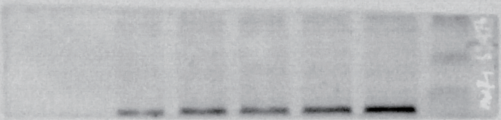

0uM Colivelin 0.5uM Colivelin 1uM Colivelin 2uM Colivelin 4uM Colivelin

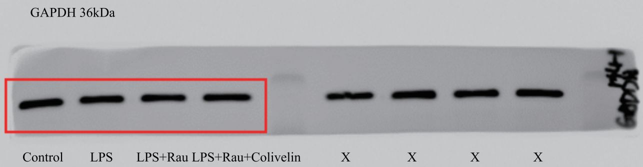
GAPDH 36kDa
Control
LPS
LPS+Rau
LPS+Rau+Colivelin
X
X
X
X

NOS2 131kDa

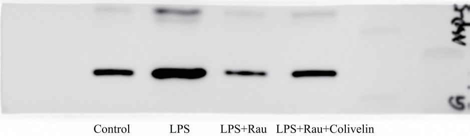

JAK2 131kD

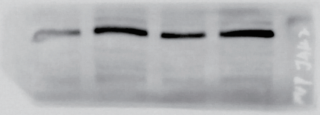

Control LPS LPS+Rau LPS+Rau+Colivelin

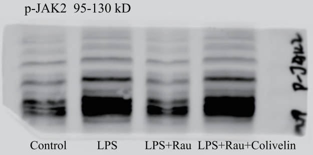
p-JAK2 95-130 kD
Control
LPS
LPS+Rau
LPS+Rau+Colivelin

p-STAT3 131kDa

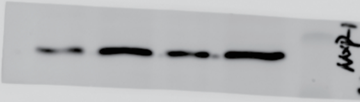

Control LPS LPS+Rau LPS+Rau+Colivelin

STAT3 88 kDa

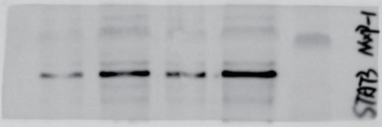

Control LPS LPS+Rau LPS+Rau+Colivelin

GAPDH 36 kDa

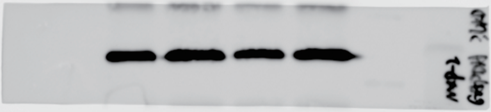

Control DSS DSS+200mg/kg/d Rau DSS+200mg/kg/d Rau+Colivelin

NOS2 131kDa

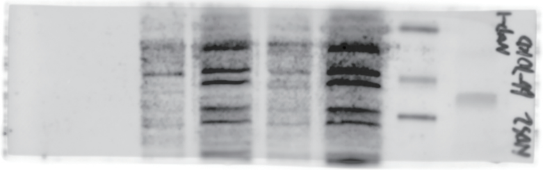

Control DSS DSS+200mg/kg/d Rau DSS+200mg/kg/d Rau+Colivelin

JAK2 131kD

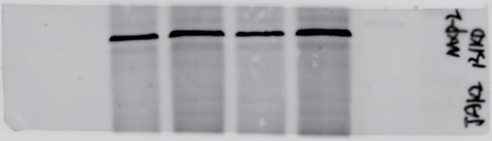

Control DSS DSS+200mg/kg/d Rau DSS+200mg/kg/d Rau+Colivelin

p-JAK2 95-130 kD

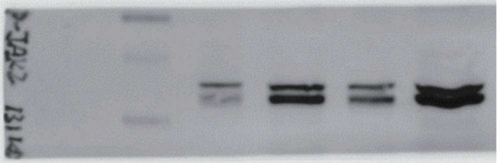

p-STAT3 80-100 kDa

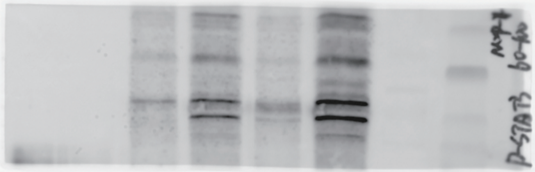

Control DSS DSS+200mg/kg/d Rau DSS+200mg/kg/d Rau+Colivelin

STAT3 88 kDa

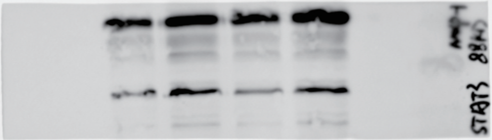

Control DSS DSS+200mg/kg/d Rau DSS+200mg/kg/d Rau+Colivelin
